# Osteoarthritis Increases Paresthestic and Akathisic Pain, Anxiety Case-ness, and Depression Severity in Patients With Parkinson's Disease

**DOI:** 10.3389/fneur.2018.00409

**Published:** 2018-06-13

**Authors:** Abdul Qayyum Rana, Abdul Rehman Qureshi, Shakib Akhter, Yameen Ingar, Ali Ayub, Ismael Abdullah, Obaidullah Madhosh, Zainab Sarfraz, Muhammad B. Rana, Ruqqiyah Rana

**Affiliations:** Clinical Neurology, Parkinson's Clinic of Eastern Toronto & Movement Disorders Centre, Toronto, ON, Canada

**Keywords:** Parkinson's disease, osteoarthritis, pain, depression, quality of life, anxiety, restless legs syndrome

## Abstract

**Objective:** Parkinson's disease (PD) patients are known to suffer from pain, anxiety, and depression, but the exact degree of association between the two is unknown. As many PD patients also suffer from physical impairments, this cross-sectional case-control study sets out to compare and determine the case-ness of pain, anxiety and depression in PD patients that suffer with or without symptomatic osteoarthritis (OA). The goal of this study, therefore, was to observe if additional pain associated with comorbid OA in PD patients is correlated with greater depression and anxiety rates. The importance of understanding the burden of pain and increased depression severity of PD and OA patients is so that they may be screened appropriately based on the symptoms, which may increase their overall quality of life.

**Methods:**This cross-sectional case-control study included 3 groups of 34 patients and 78 healthy age and gender-matched control participants. PD patients with symptomatic OA (PD+OA), PD patients without symptomatic OA (PD), patients with symptomatic OA but no PD (OA), and healthy control participants (Control). A PD patient group with Restless Legs Syndrome (PD+RLS) of 27 patients was also included. All participants completed questionnaires to assess for pain, depression, and anxiety.

**Results:**PD+OA and PD patients had worsened depression severity and were more likely to report anxiety and depression case-ness than OA patients. PD+OA patients were more likely to complain about paresthestic and akasthisic pain, but less likely to complain about aching pain compared to PD patients and OA patients. PD+OA patients were more likely to have greater pain severity, and were more likely to report radiating and sharp pain than PD+RLS patients. PD+OA patients were also more likely to report higher depression case-ness than PD+RLS patients.

**Conclusion:**PD with OA seems to be linked with specific pain characteristics (akathisia and paraesthesia) as well as heightened overall pain severity and pain interference in comparison to OA alone, PD alone and PD with RLS. PD is also correlated with depression severity and anxiety case-ness in OA when compared to the OA alone, PD alone and PD with RLS.

## Introduction

PD is characterized by not only motor symptoms but also non-motor symptoms including dementia, anxiety, and apathy (1, 2). Of the more common non-motor symptoms in PD are pain and depression, both of which contribute to a lower quality of life (3–6). Pain is found twice as often in PD patients than in a healthy control group (7–9). According to Brandt-Christensen et al., the use of antidepressant drug therapy in PD patients was 3.98 times higher than in healthy controls (10). However, non-motor symptoms are undertreated due to lack of awareness by the patient and physicians alike (5, 11, 12).

Numerous studies have analyzed the relationship between pain and depression in PD (4, 7, 10, 13–15). However, the association between the two remain unclear. As a result, the treatment of PD is inadequately met (11). Studies by Ehrt et al. (4) and Nilsson et al. (13) on hospitalized patients with advanced PD found that these patients had greater depression rates than those with a similar functional disability from other illnesses, such as symptomatic osteoarthritis (OA) or diabetes mellitus. Overall, the majority of studies have found significant depression rates in PD patients, indicating depression as something intrinsic to PD (7, 10, 13). However, the results are inconsistent, as the findings presented in both experiments account for a small proportion of variability in PD and OA patients (14, 16). Although PD exhibited greater depression than OA in the aforementioned studies, no study to our knowledge has studied depression in patients with PD and OA comorbidity relative to PD and healthy samples.

Furthermore, many studies have analyzed the relationship between anxiety in PD patients (17–19). Akin to the studies conducted on depression and pain in PD, the direct association between anxiety and PD remains unclear. In the study conducted by Pontone et al. (17) and Sagna et al. (18), a higher case-ness of anxiety and anxiety related disorders was present in the PD cohort. However, the case-ness of anxiety along with other conditions, such as depression, OA and increased pain severity in PD patients has yet to be explored.

Restless Legs Syndrome (RLS) is a neurological sleep disorder which results in an essential feeling to move one's legs as a result of uncomfortable sensations and motor restlessness (20). In previously conducted studies, many PD patients with RLS have reported different types of pain, ranging in severity (21, 22). As observed by Allan and colleagues (21), PD patients that were diagnosed with RLS experienced more limb paresthesia and reported more painful cramps. Although studies analyzing pain in PD patients with RLS have been conducted, a comparison between PD patients with RLS vs. OA patients with PD has not been studied.

This cross-sectional case-control study expands on previous reports in order to evaluate a possible relationship between PD and OA comorbidity and non-motor symptoms (i.e., neuropsychiatric and sensory disturbances) in this condition. The study also analyzes the relationship between the degree of physical impairment (pain) and the degree of depression in PD in patients with PD with RLS, only PD, or only OA. OA is considered the most common disorder of the musculoskeletal system, with higher rates of prevalence in the elderly (5). The comorbidity of OA with depression has been reported. According to one study, depressive symptoms in OA patients lead to greater pain perception and specific pain (5). However, there is no current study that has examined both depression and pain in relation to PD and OA.

The objectives of this study are (i) to determine if there is a relationship of PD in OA with anxiety, pain and depression, and (ii) to determine the association of anxiety, pain and depression between two PD comorbid populations of PD with RLS and PD with OA.

## Methods

### Participant recruitment

The study comprised of 34 PD patients with symptomatic OA (PD+OA), 34 PD patients without symptomatic OA (PD) and 34 patients with symptomatic OA but no PD (OA). Patients with dementia or atypical parkinsonism were not included in the study. A control group (Control) of healthy individuals matching the 78 cases by age and gender was also included. There were 17 males and 17 females in each group. Mean age of the cases together was 71.2 ± 10.5 years, with the PD+OA group aged 75.3 ± 8.32 years, the PD group aged 64.8 ± 11.7 years, and the OA group aged 73.6 ± 8.24 years. Mean age for the control group (Control) was 67.7 ± 12.5 years. PD patients with Restless Legs Syndrome (RLS; PD+RLS) were also included in this study (*n* = 27, mean age ± SD = 69.7 ± 11.0 years), comprising of 16 males and 11 females. All participants visited a community PD and movement disorders clinic from June 2011 to June 2012 for questionnaire assessment via semi-structured interviews followed by a neurological examination. All participants were randomly selected for inclusion into the study. PD patients visited the clinic routinely (at least 1–2 times) during the study period. The control participants originated from the same demographical region as the PD patients, accompanied the PD patients to their routine neurological assessments, were not biologically related to the PD patients, and had no past history of sleep, cognitive or movement disorders.

### Participant assessment

The HADS (Hospital Anxiety and Depression Scale) has a depression section (HADS-D) and an anxiety section (HADS-A), which were collectively used to assess depression severity, depression case-ness, anxiety severity and anxiety case-ness (23). If a participant had scored a HADS-D score of 8 or greater, then they were deemed positive for depression case-ness. Similarly, if a HADS-A score was 8 or greater for a participant, then they would be positive for anxiety case-ness (24). The Brief Pain Inventory (BPI) was used to assess pain interference, pain severity and pain characteristics. Pain interference was further assessed using two quality of life (QOL) dimensions of the BPI. The first QOL dimension is REM, which assessed pain interfering with relationships, enjoyment of life, and mood. The second QOL dimension is WAW, which assessed pain interfering with walking ability, general activity and normal work activities. (25) The patients with OA were diagnosed by their family physicians before the study, through the diagnostic criteria they deemed necessary (26). The diagnosis of PD was made using the UK brain bank criteria (27). The diagnosis of RLS was made using the RLS diagnostic criteria (21). All participants had given their informed and written consent in accordance with the Declaration of Helsinki. The study was reviewed and approved by the local ethics board of the Rouge Valley Health System.

### Statistical analysis

The clinical variables are defined as the severity and case-ness scores from the HADS, along with the severity and interference measures from the BPI. The demographical variables are age, gender, and the PD-specific parameters of disease duration, UPDRS-III score, age of diagnosis, and H&Y stage. Significant differences between the four groups (PD+OA, PD, OA, and Control) with respect to the clinical variables and age was assessed through the Kruskal–Wallis 1-way ANOVA using Conover's *post hoc* method and the Benjamin–Hochberg procedure for *p*-value adjustment. PD-specific parameters were compared between the PD groups (PD+OA and PD) using the Mann–Whitney *U* test. An exploratory factor analysis (EFA) informed by eigenvalues and scree plots was conducted to investigate correlations between all clinical and demographical variables. Multiple regression was conducted to determine if the demographical variables correlate with the clinical variables. An ordinal logistic regression analysis adjusted for age was conducted to determine whether PD, OA or PD and OA comorbidity was predicted by any of the pain characteristics of the BPI, which were: radiating, aching, dull, tension, sharp, boring, shooting, throbbing, burning, stabbing, miserable, cramping, paresthestic and akathisic. A binary logistic regression analysis was conducted to determine whether PD with OA or RLS comorbidity was predicted by any of the clinical variables. All reported values were stated to at most 2 significant figures. The significance threshold for all analyses was set to *p* = 0.05.

## Results

PD+OA patients scored higher on HADS-D severity than OA patients and Control participants by 37 and 43 mean rank points (*p* = 0.00010 and *p* < 0.0001). PD patients scored 28 and 35 mean rank points higher than OA patients and Control participants on HADS-D severity (*p* = 0.00017 and *p* = 0.002). PD+OA patients scored 24 points higher on WAW, and 25 points higher on BPI interference compared to Controls (*p* = 0.027 and *p* = 0.016). PD+OA patients scored higher than OA and PD patients on BPI interference and BPI severity, though it was not statistically significant (*p* = 0.057, *p* = 0.057, *p* = 0.10 and *p* = 0.05). PD+OA patients had higher HADS-D case-ness by 15 and 11 more counts than Control individuals and OA patients (*p* = 0.001 and *p* = 0.005). PD patients had higher HADS-D case-ness by 12 and 8 more counts than Control individuals and OA patients (*p* = 0.001 and *p* = 0.009). PD+OA patients also had higher HADS-A case-ness by 9 and 10 more counts than OA patients and Control individuals (*p* = 0.012 and *p* = 0.003), (Table 1).

**Table 1:** Descriptive statistics of a sample of Parkinson's disease patients with osteoarthritis (PD+OA), Parkinson's disease patients (PD), osteoarthritis patients (OA), and control participants (Control)^*^.

	**PD+OA (*n* = 34)**	**PD (*n* = 34)**	**OA (*n* = 34)**	**Control (*n* = 34)**	***P***
**DEMOGRAPHICAL VARIABLES**					
Age	75.3 ± 8.32	64.8 ± 11.7	73.6 ± 8.24	67.7 ± 12.5	0.00043^A^ 0.0087^C^0.0078^E^
Age of diagnosis	71.4 ± 8.88	61.4 ± 12.4	–	–	0.0013^A^
Disease duration	3.94 ± 3.54	3.31 ± 3.84	–	–	NS
UPDRS-III score	26.4 ± 6.45	20.2 ± 7.18	–	–	0.001^A^
H&Y stage score	2.53 ± 0.627	2.21 ± 0.428	–	–	0.016^A^
**CLINICAL VARIABLES**					
BPI severity	83.2 ± 6.42	62.6 ± 5.51	66.4 ± 5.99	61.7 ± 6.03	NS
BPI interference	83.8 ± 6.19	65.7 ± 5.44	65.7 ± 6.12	58.8 ± 5.35	0.016^C^
REM	81.8 ± 6.06	66.0 ± 5.54	65.3 ± 5.76	61.0 ± 5.28	NS
WAW	83.0 ± 6.19	64.6 ± 5.30	67.0 ± 6.17	59.5 ± 5.21	0.027^C^
HADS-D severity	90.6 ± 5.71	82.0 ± 6.41	54.0 ± 6.45	47.4 ± 5.49	0.00010^B^ < 0.0001^C^0.002^D^0.00017^E^
HADS-A severity	80.8 ± 6.66	71.2 ± 7.72	60.1 ± 6.32	61.9 ± 5.80	NS
HADS-A case-ness	15 (44.1%)	14 (41.2%)	6 (17.6%)	5 (14.7%)	0.012^B^0.003^C^
HADS-D case-ness	19 (55.9%)	16 (47.1%)	8 (23.5%)	4 (11.8%)	0.005^B^0.001^C^0.001^D^0.009^E^
**PARKINSONIAN MEDICATIONS**					
Carbidopa-levodopa	24 (71%)	25 (74%)	–	–	NS
Pramipexole	12 (35%)	3 (8.8%)	–	–	NS
Amantadine	11 (32%)	7 (21%)	–	–	NS
Levodopa-benserazide	1 (2.9%)	2 (5.9%)	–	–	NS
Ropinirole	2 (5.9%)	0 (0.0%)	–	–	NS
Rasagiline	3 (8.8%)	3 (8.8%)	–	–	NS
Selegiline	1 (2.9%)	0 (0.0%)	–	–	NS
Entacapone	1 (2.9%)	4 (12%)	–	–	NS
Trihexyphenidyl	1 (2.9%)	1 (2.9%)	–	–	NS
**ANTI-DEPRESSANT MEDICATIONS**					
Lithium	1 (2.9%)	0 (0.0%)	–	–	NS
Trazodone	1 (2.9%)	0 (0.0%)	–	–	NS
Mirtazapine	2 (5.9%)	1 (2.9%)	–	–	NS
Citalopram	1 (2.9%)	0 (0.0%)	–	–	NS
Venlafaxine	1 (2.9%)	1 (2.9%)	–	–	NS
Sertraline	0 (0.0%)	2 (5.9%)	–	–	NS
Quetiapine	0 (0.0%)	2 (5.9%)	–	–	NS
Amitriptyline	0 (0.0%)	1 (2.9%)	–	–	NS
Escitalopram	0 (0.0%)	1 (2.9%)	–	–	NS

*A PD+OA vs. PD;

B PD+OA vs. OA;

C PD+OA vs. Control;

D PD vs. Control;

E PD vs. OA. HADS, Hospital Anxiety and Depression Scale;

*BPI, Brief Pain Inventory; UPDRS-III, Unified Parkinson's Disease Rating Scale – Section III: Motor Component; H&Y, Hoehn and Yahr Stage; NS, not statistically significant. Demographic and clinical variables are expressed as mean ± SE. Clinical variables are expressed as mean ± SE rank points*.

PD+OA patients were older than PD patients by 11 years (*p* < 0.00043) and Control individuals by 8.1 years (*p* = 0.0087). OA patients were also older than PD patients by 8.8 years (*p* = 0.0078). PD+OA patients had a higher age of diagnosis of PD than PD patients by 10 years (*p* = 0.0013). PD+OA patients also had higher UPDRS-III scores and H&Y stage scores, surpassing the PD patients by 6.2 and 0.32 points (*p* = 0.001 and *p* = 0.016), respectively, (Table 1). However, multiple regression revealed that only age (*p* = 0.036) and age of diagnosis (*p* = 0.02) correlated with HADS-A severity. All other correlations of demographic variables with clinical variables were statistically insignificant.

The EFA revealed five factors from the clinical and demographic variables (Table 2). Factor 1 accounted for 27% of the variance in data, with BPI scores showing the highest factor loading (IFL ≥ 0.91). Twenty percent of the variance was due to Factor 2, with both HADS-A and HADS-D scores having the strongest loading (IFL ≥ 0.80). Factor 3 was most strongly represented by age and age of diagnosis, accounting for 16% of the variance in data (IFL ≥ 0.94). Factor 4 emphasized PD-specific parameters which accounted for 15% of the variance in data (IFL ≥ 0.78), and Factor 5 emphasized gender responsible for 9% of the variance in data.

**Table 2:** Factor structures of the five factors for the clinical and demographical variables*.

**Factor**	**Item**	**Item factor loading (IFL)**
Factor 1	**Percent Variance: 26.7**
	BPI severity	0.918
	BPI interference	0.962
	REM	0.922
	WAW	0.940
Factor 2	**Percent Variance: 19.6**
	HADS-A severity	0.806
	HADS-D severity	0.863
	HADS-A case-ness	0.758
	HADS-D case-ness	0.773
Factor 3	**Percent Variance: 15.7**
	Age of diagnosis	0.989
	Age	0.941
Factor 4	**Percent Variance: 15.0**
	UPDRS-III score	0.801
	H&Y score	0.782
	Disease duration	0.829
Factor 5	**Percent variance: 9.05**	
	Gender	0.868

* BPI, Brief Pain Inventory;

*HADS, Hospital Anxiety Depression Scale; UPDRS-III, Unified Parkinson's Disease Rating Scale – Section III: Motor Component; H&Y, Hoehn and Yahr Stage*.

An ordinal logistic regression analysis adjusted for age found that PD+OA patients were 6.0 times more likely to report paresthestic pain and 3.9 times more likely to report akathisic pain compared to PD patients and OA patients (*p* = 0.027 and *p* = 0.001). PD+OA were 36% less likely to report aching pain compared to PD patients and OA patients (*p* = 0.028). PD, OA, and PD and OA comorbidity was not significantly predicted by any of the other BPI pain characteristics, (Table 3). PD+RLS patients had age of diagnosis at 65.3 ± 12.3 years and disease duration of 4.00 ± 5.40 years. UPDRS-III score was 23.7 ± 6.71 and H&Y stage score was 2.33 ± 0.537 for the PD+RLS patients. Since PD+RLS patients were significantly younger in age than PD+OA patients (*p* = 0.034), the binary logistic regression analysis was adjusted for age. PD+OA patients were 30 times more likely to report radiating pain (*p* = 0.042), 48 times more likely to report sharp pain (*p* = 0.015), and 1.5 times more likely to report higher BPI pain severity (*p* = 0.016), and 68 times more likely to report greater HADS-D depression caseness (*p* = 0.049) compared to PD+RLS+ patients. PD+OA patients were also 97% less likely to report dull pain (*p* = 0.016) compared to PD+RLS+ patients. PD with OA or RLS comorbidity was not significantly predicted by any other variables (Table 4).

**Table 3:** The relationship of PD, OA, and PD and OA comorbidity with pain characteristics^*^.

**Predictor**	**Exp(β)**	***P***
Radiating	1.75	NS
Aching	0.364	0.028
Dull	1.04	NS
Tension	1.61	NS
Sharp	0.779	NS
Boring	3.26	NS
Shooting	0.648	NS
Throbbing	1.56	NS
Burning	2.09	NS
Stabbing	1.70	NS
Miserable	1.30	NS
Cramping	0.951	NS
Paresthestic	3.91	0.027
Akathisic	6.02	0.001
Nagelkarke *R*^2^	0.303	< 0.01

* Model adjusted for age. PD, Parkinson's disease;

*OA, osteoarthritis; PD+OA, Parkinson's disease and osteoarthritis; Exp(β), odds ratio; NS, not statistically significant*.

**Table 4:** The relationship of PD and OA comorbidity or PD and RLS comorbidity with measures of depression, anxiety, pain, and QOL.

**Predictor**	**Exp(β)**	***P***	**Predictor**	**Exp(β)**	***P***
**BPI Pain characteristics**			**BPI Pain characteristics**
Radiating	29.8	0.042	Parethestic	2.13	NS
Aching	0.386	NS	Akathisic	0.408	NS
Dull	0.026	0.016	**HADS**
Tension	2.15	NS	HADS-A depression case-ness	67.8	0.049
Sharp	47.5	0.015	HADS-D depression severity	1.75	NS
Boring	~0	NS	HADS-D anxiety case-ness	1.52	NS
Penetrating	0.797	NS	HADS-D anxiety severity	0.969	NS
Shooting	0.217	NS	**BPI**
Throbbing	3.44	NS	BPI pain severity	1.52	0.016
Burning	13.1	NS	BPI pain interference	0.844	NS
Stabbing	0.408	NS	REM	0.941	NS
Miserable	10.7	NS	Nagelkarke R^2^	0.625	< 0.05
Cramping	0.497	NS		

*Model adjusted for age. WAW was entered into the analysis but removed by the regression model due to its negligible effect on PD with OA or RLS comorbidity. PD, Parkinson's disease; BPI, Brief Pain Inventory; HADS, Hospital Anxiety and Depression Scale; OA, osteoarthritis; RLS, Restless legs syndrome; Exp(β), odds ratio; NS, not statistically significant*.

## Discussion

We assessed the non-motor symptoms of PD of pain and depression in association with symptomatic OA (OA). One of the unique features of this study design was analyzing specific types of pain in patients with both PD and OA. The main findings of this study were that the severity of depression and the case-ness of pain severity, radiating pain, sharp pain and anxiety were higher in the PD+OA cohort than in the PD patients, OA patients, PD+RLS patients and the controls. Interestingly, the analysis showed complaints of aching, dull and paresthestic pain being unusually higher in patients with PD without OA compared to those with PD and OA. Furthermore, the case-ness of depression in the PD+OA patients was not significantly greater than the PD patients, as indicated by the HADS-D score. This is similar to the large cross-sectional study of DoPaMip, where PD patients with pain had similar depression rates as those PD patients without pain, despite its severity (9). Hence, regardless of the accumulating pains of PD patients with comorbid OA, such subjects were found to be as significantly depressed as those PD patients without comorbid OA pain. This suggests that despite the pain level in PD patients, PD itself may carry the potential for greater depression in comparison to OA patients and Control individuals.

In a recent study assessing the relationship between pain, OA, and depression in the older adults, it has been found that pain intensity was correlated with depression due to the confounding effect of activity restriction (28). In line with this finding, Gotham et al.'s (14) study found no difference in the Beck Depression Inventory scores between the PD and OA patients. The findings of both studies are in contrast with the present study, as only 23.5% of the OA group exhibited depression case-ness. The opposite findings of this study may be attributed to different populations being studied, in which more severe cases of OA were under investigation. Our subjects may have had milder conditions of OA. Moreover, the discrepancy in our results relative to past studies may be from the location of OA, which may dictate how potentially influential or restrictive the intensity of pain is to activities of daily living.

Many previous studies have also shown similar results to the study when analyzing PD and depression (8, 13, 18). In a case-control study analyzing the case-ness of pain in PD subjects and healthy controls, it has been found that the PD cases suffered greater frequencies of pain in comparison to the controls that lacked dystonic pain (4, 29). Nilsson et al. (13) found that regardless of the age of PD onset or gender, the probability of a diagnosis for depression was increased in PD patients in comparison to their OA patients. Only a few studies found inconsistent results where there was no difference between healthy elderly individuals or OA patients (16).

Furthermore, many previous studies did not account for age when analyzing data. However, in this study, since PD+RLS patients were significantly younger than PD+OA patients, the variability for age was accounted for in our analyses. This ensures that there was no confounding factor and thus limits the potential risk of bias within the groups.

This study has several limitations. First, the groups were not divided according to depression case-ness to evaluate the effects of pain disability and OA case-ness. Since the patients had different degrees of pain severity within multiple joints and might have taken different medications, which could have affected the results. Thus, it was not possible to divide patients into various groups of OA. Also, given the correlative nature of depression to many factors such as age and the severity of parkinsonism, further studies should control these factors when assessing depression in PD and OA. In addition, the diagnosis of OA in patients participating in the study was done by their family physicians, based on the relevant clinical criteria as outlined by Altman (26). Moreover, as the patients were on a combination of multiple different medications due to the complex nature of PD, specific doses of medications were not accounted for. Lastly, despite a higher association between non-motor symptoms of pain and depression in the PD+OA cohort, the association between PD, functional impairment, and depression are correlational and not causative due to the cross-sectional nature of the research. One study has found that those diagnosed with depressive disorders were more likely than those with OA to be diagnosed with PD later in life (30). Future longitudinal studies are recommended to better clarify the relationship between pain, depression, and PD. However, a unique feature of this study was comparing PD patients with OA to not only PD patients without OA, but also to healthy controls with OA, and PD patients with RLS. While assessing pain and depression is a significant aspect of living with PD, it is also important to understand the effect on OA patients as well.

Additionally, in order to enhance studies regarding pain, anxiety and depression in PD and OA, future studies should utilize advanced methodologies. Such advanced methodologies should include medical imaging techniques when identifying biomarkers in the brain. In particular, future research should utilize different functional magnetic resonance imaging (fMRI) techniques such as diffusion tensor imaging (DTI) which analyzes the structure of brain tissues or arterial spin labeling (3D-ASL) which uses labeled arterial blood water in order to measure cerebral blood flow (31). Both DTI and 3D-ASL techniques can be utilized in order to mark the differences between PD patients and healthy controls. Similarly, in a study conducted by Xu et al. (32), the severity of vascular parkinsonism and PD was investigated using imaging with 18^F^-fluorodeoxyglucose (18^F^-FDG) through determining different patterns of glucose metabolism and abnormal functionality in neurodegenerative diseases. In future studies, 18^F^-FDG imaging should be utilized in determining the differences in PD patients vs. PD and OA patients, as well as healthy controls. Additionally, the use of blood sampling to identify biomarkers relevant in the pathogenesis of neurodegenerative diseases is recommended (33, 34). Wang et al. (33) showed that the plasma levels of two biomarkers (cystatin C and high-density lipoprotein) were abnormal in patients with Alzheimer's disease and vascular dementia. Similarly, in a study conducted by Chen and colleagues (34), levels of the biomarkers homocysteine and uric acid were shown to be lower in patients with multiple system atrophy. Previous research has not been specific to PD, and therefore further studies should evaluate the aforementioned biomarkers in PD patients to determine if they are effective markers for PD. Overall, future studies investigating PD, OA, pain and depression should utilize advanced MRI techniques and blood sampling measurements in order to enhance the research methodologies.

In conclusion, the treatment of pain and depression should a fundamental component in the standard care of PD due to the effect it has on health-related quality of life (6). Implementation through a general practitioner's evaluation as well as patient education and an integrated holistic approach to care is recommended. Adequate treatment may potentially help to important non-motor symptoms, and consequently, decrease patient's dependence, burden of pain, and depression. Future robust and methodologically sound research may establish a potentially causal relationship between PD, RLS, depression and its interplay with OA.

## Author contributions

AR, RR, ZS, and MR: substantial contribution to the conception of the work, drafting the work or revising it critically for important intellectual content, final approval of the version to be published, agreement to be accountable for all aspects of the work in ensuring that questions related to the accuracy or integrity of any part of the work are appropriately investigated and resolved. AQ: conception, analysis, acquisition and interpretation of the work, drafting the work or revising it critically for important intellectual content, final approval of the version to be published, agreement to be accountable for all aspects of the work in ensuring that questions related to the accuracy or integrity of any part of the work are appropriately investigated and resolved. YI, AA, OM, and IA: interpretation of the work, drafting the work or revising it critically for important intellectual content, final approval of the version to be published, agreement to be accountable for all aspects of the work in ensuring that questions related to the accuracy or integrity of any part of the work are appropriately investigated and resolved. SA: substantial contribution to the conception of the work, such as piloting and conducting a new set of statistical analyses and reinterpreting the data, entirely redrafting the manuscript and revising it critically for important intellectual content, final approval of the verison to be published, and agreement to be accountable for the study.

### Conflict of interest statement

The authors declare that the research was conducted in the absence of any commercial or financial relationships that could be construed as a potential conflict of interest.
